# On Instinct

**Published:** 1841-01

**Authors:** 


### ART. IV.

1. *On Instinct.* By W. P. ALISON, M.D., &c. (*Cyclopædia of Anatomy.* Part XIX.)—*London*, 1840. Royal 8vo, pp. 29.

2. *The Philosophy of Instinct and Reason.* By J. STEVENSON BUSHNAN, M.D., F.L.S.—*Edinburgh*, 1837. *With Eight Lithographic Plates.* 12mo, pp. 316.

3. *On the Habits and Instincts of Animals.* By WILLIAM SWAINSON, F.R.S., &c. (*Cabinet Cyclopædia.*)—*London*, 1840. *With Ninety-one Woodcuts.* Small 8vo, pp. 375.

THE designation *instinct*, it is well observed by Dr. Alison at the commencement of his able article, "is often applied to the mental acts of the lower animals, as if it were truly applicable to the whole of those acts, but a little consideration will show, *first*, that this word in its more approved and correct acceptation, is applicable only to a *part* of the mental operations which may be inferred from the observation of the actions and habits of animals; and, *secondly*, that in this restricted sense the term is applicable to a part of the operations of the human mind itself, and that the subject of instinct cannot be thoroughly understood, unless information regarding it is sought in the consciousness of our own minds, as well as in the observation of other living beings. The study of this subject is therefore equally important as a part of natural history, of mental philosophy, and of human physiology, and is a good illustration of the necessity of this latter science being based on the observation and generalization of the laws and conditions of vital action throughout the whole extent of the animal creation." Believing that, on these accounts, our readers will feel interested in the subject, we shall offer them a short account of Dr. Alison's views, and some remarks of our own thereupon.

The term instinct, according to Dr. Alison, stands in opposition, not to the *will* but to the *reason* of man. "The most correct expression of the difference between an action prompted by instinct and one prompted by reason is, that in the first case the will acts in obedience to an impulse which is directly consequent upon certain sensations or emotions felt or remembered; in the last, it acts in obedience to an impulse which results from acts of reasoning and imagination." (p. 3.) With the general view which this proposition is intended to enunciate, we fully accord, but we must differ from Dr. Alison in regard to the opinion that the *will* is concerned in the production of *instinctive* actions. It will be remembered, that on a former occasion we made use of this term to include all those actions "which are performed in direct respondence to an external stimulus," (Vol. V. p. 491;) and we accordingly give it a much more extended application than does Dr. Alison, who restricts it to what we deem the highest class of such actions. But even using it in *his* sense, we cannot regard instinctive actions as produced through the will, *when instinct is acting alone.* In a great majority of cases, the instincts of man suggest motives to his judgment, and the resulting action is voluntary, although first stimulated by the instinctive propensity. But, in animals whose instincts predominate over the reasoning powers (the contrary being the case in man), we are of opinion that these instincts act *primarily* on the motor nerves. The case seems to us exactly parallel with that of the emotions, on some of which the instinctive propensities border very closely. There is no question that the emotions can act on the muscular system, through the efferent nerves, independently of the will, and even in opposition to it; and that they operate through a distinct set of nervous filaments appears proved by the fact that the muscles may be paralyzed to one set of impulses, and not to the other. But, though the emotions *can* act immediately on the motor nerves, it is rarely that they do so in man, for they usually, that is, when only moderately excited, influence the current of thought, supply motives to the judgment, and therefore operate through the will. Physiologically speaking, the two channels are perfectly distinct, and the question is, to which we are to refer the instinctive actions "in which the impulse is directly consequent upon sensations or emotions." We maintain that it is most correct to refer them to the involuntary class, and to apply the term *will* only to that action of mind upon muscle which "results from acts of reasoning and imagination." Perhaps it may be said that this is a question of words only, involving nothing more than the signification of the term *will*, which may be extended or not at the pleasure of the writer. But though it may be so to the metaphysician, it is not to the physiologist, who seeks to allocate in the nervous system the different psychical powers, of which, as a whole, it is unquestionably the instrument.

We shall, for the sake of convenience, employ the term instinct for the present in Dr. Alison's sense; excluding from the group of actions we formerly associated under it all save those in which *sensation* participates. The question now arises whether the lower animals are so formed that *all* their actions are instinctive, that is, are performed in direct respondence to a stimulus acting through the sensorium; or whether any of them involve the operation of reasoning powers. According to Dr. Alison, the latter is the occasional exception; the former the general rule. Still he admits that the examples which he cites, and which we forbear to quote because previously in print, "leave no reasonable ground for doubt that, on certain subjects at least, some animals are capable of short and simple processes of reasoning or of imagination, which appear to imply the perception of general truths; and that the difference between the operations of their minds and ours in that respect is one of degree only, not of kind." That a deficiency of the power of forming *general notions*, and a limitation of their minds to particulars, and hence an incapacity for that adaptation of means to ends which implies a knowledge of the effect of these means founded upon general principles, is characteristic of the lower animals, we readily allow; but we do not think that Dr. Alison has given them quite as much credit for reasoning powers as some of them deserve. The following circumstance, communicated to us by several eye-witnesses, is, we think, a very good illustration of the reasoning power of brutes. Some horses kept in a paddock were supplied with water by a trough which was occasionally filled from a pump—not, however, as often as the horses seem to have wished; for one of them learned (*suâ sponte*) to supply himself and his companions by taking the pump-handle between his teeth and working it with his head. The others, however, appear to have been less clever or more lazy, and finding that this one had the power of supplying their wants they would teaze him, by biting, kicking, &c. until he had pumped for them, and would not allow him to drink until they were satisfied. Now it may be said that this was a mere act of imitation; but we think the contrary. When brutes *imitate* the actions of man, it is by a movement of the corresponding parts of the body, as when a monkey plays a knife and fork and takes his glass of wine like "any chrisom child." We can scarcely avoid the belief that this clever horse had formed a *general notion* that the action of working the pump-handle up and down would cause the flow of water into the trough, and that he adapted his means to the end in view with a sagacity which would have done credit to many a human being. If, following him, the other horses had done the same, we should have ascribed it to imitation *in them;* just as when sheep follow their leader through a gap he has discovered in a hedge.

Another instance, which also exemplifies the formation of general notions, we shall relate on the authority of an eye-witness. A wren built its nest in rather a dangerous situation in the quarries of Penrhyn, so as to be liable to great disturbance from the occasional explosions. It soon learned, however, to quit its nest and fly to a little distance, on the ringing of the bell which warned the workmen. This was noticed, and demonstrated to visitors, so that the poor wren suffered many needless alarms. It at last learned, however, that the first general notion it had formed—of the ringing of the bell being followed by an explosion—was liable to exceptions, and it formed another more correct. For it was observed after a time that the wren did not leave its nest unless the ringing of the bell was followed by the moving away of the workmen. Dr. Alison further states that "none of those still more general or abstract notions which continually suggest themselves to the human mind in the course of the operations that are excited by the senses, such, for example, as space, time, number, power, &c. are indicated by any manifestations we see of the mental acts of the lower animals." In this, too, we scarcely think that he does them justice. Many instances are on record which show such a remarkable knowledge of the passage of time, and the period of recurrence of particular epochs, that we can scarcely avoid the conclusion that some such general notions exist. At least we see as much reason for inferring them from their actions as we should from the actions of man, apart from our own consciousness. A case of this sort, as interesting as any that we have heard of, has fallen within our own experience. A number of young ladies, pupils in a boarding-school, are accustomed to go daily into their playground at a quarter past twelve, and there to eat their luncheon. They return to the schoolroom after about a quarter of an hour, leaving, of course, a good many crumbs on the ground behind them. The sparrows in the neighbourhood have learned to expect these, and for a quarter of an hour before the daily "run out," as well as during that period, they may be seen congregated on the walls of the playground ready to pounce down as soon as the pupils have left it. Their punctuality is remarkable. But on Sundays, when, on account of an earlier dinner-hour, no luncheon is eaten, the birds do not expect it, for no congregation of them takes place. The case would be less remarkable if the birds only collected *during* the time when the pupils are in the ground, as the omission on Sundays would then be accounted for by the want of the usual signal; but they assemble regularly on week-days *before* any such signal is given, as we have ourselves witnessed many times.

We are disposed to believe, then, that in the higher classes of those commonly denominated the lower animals, reasoning powers exist rather inferior to those of men in degree than differing in kind; and that, as we ascend the scale, the instincts are gradually subordinated to them; so that, in the highest among them in point of intelligence, the general conduct is rather governed by the will, acting in obedience to intellectual operations (which may be originally stimulated by the instinctive propensity) than in direct respondence to the stimulus of sensation. The dog and the elephant are usually regarded as the most intelligent of the lower animals; but it may be questioned whether their intelligence is *really* greater than in other mammalia, the quadrumana for example; or whether it does not appear so to us in consequence of their instinctive attachment to man and the accommodation of their actions to his. Of the *reasoning* power of the elephant we shall adduce an interesting example from the work of Mrs. Postans on British India, which will be probably new to most of our readers. It was communicated to the authoress by the owner of the animal, an officer in the Bengal service. This gentleman "possessed a handsome elephant, which he was accustomed to see fed with a certain allowance of grain daily. Business requiring his absence, he confided the care of his favorite to a worthless keeper, who, in the interim, stole and appropriated a large portion of the grain intended for the elephant's use. The poor animal daily grew more spare and feeble, missing at his usual feeding-time the abundant feast supplied by his kind and generous master. My friend returned, hastened to his stable, observed the emaciated state of his favorite, and having had no previous reason to suspect the honesty of the servant, was at a loss to discover the cause of the evident alteration. The poor elephant, delighted at his master's return, trumpeted his welcome, raised his trunk as a salaam, and moved about, affording, in his mute but expressive manner, every demonstration of joy. His feeding-time approached, and the full allowance of grain was placed at his feet by his dishonest and cruel keeper. The elephant, satisfied of his *master's* attention, industriously separated it into two distinct heaps, and having eagerly devoured the one left that which remained and quietly walked to the opposite side of the stable. The truth, thus conveyed by the gestures of the intelligent brute, flashed upon the mind of his master. The keeper, on being accused of the theft, and finding his unworthiness exposed, fell at the feet of his employer, acknowledging the aggression." Now, we would ask, could any mute human being more justly reason or more cleverly adapt his means to the end in view? "The native keepers of elephants," adds our authoress, "will not allow that the animals are influenced by the passion of fear, but declare their obedience to be an impulse of gratitude, and believe them to possess the reason peculiar to human nature." One more instance of rationality, with a degree of self-control which it may be wished that every human being would imitate, we shall quote from Dr. Davy, (Anatomical and Physiological Researches, vol. i., p. 884,) who himself witnessed it. The animal had a deep abscess in the back, which it was necessary to lay open in order to effect a cure. "He was kneeling down, for the convenience of the operator, not tied, his keeper was at his head. He did not flinch, but rather inclined towards the surgeon, uttering a low suppressed groan. He seemed conscious that what was doing was intended for his good; no human being similarly situated could have behaved better. I think it right thus to record this instance, which I witnessed myself, of this animal's (may I call it) reflecting power and conduct, which it is difficult to consider otherwise than rational. And so confident were the natives that he would behave as he did that they never thought of tying him."

It can scarcely be questioned, we think, that the *general* character of the intellectual powers of the lower animals is such as Locke represented it; a very limited enjoyment of the faculty of *abstraction*, by which the mind is enabled to single out the different qualities or relations of the individual objects of sense, and make them the subject of abstract thought, by which general notions are formed.

"The two noblest and most characteristic of the faculties of the human mind, as defined by logicians," remarks Dr. Alison, "those of reasoning and imagination (the latter of which is truly applicable to every kind of *contrivance* or adaptation of means to ends of which we are capable), evidently imply the existence of a mental power of forming and dwelling on general ideas, which are equally applicable to many individual cases; and if animals possessed this latter power, we might confidently expect to see them exhibit indications of the two former. Why is it, for example, that the monkeys, who have been observed to assemble around the fires which savages have made in the forests, and been gratified by the warmth, have never been seen to gather sticks and rekindle them when expiring? Not, certainly, because they are incapable of understanding that the fire which warmed them formerly will do so again, but because they are incapable of abstracting and reflecting on that *quality* of wood and that *relation* of wood to fires already existing, which must be comprehended, in order that the action of renewing the fire may be suggested by what is properly called an effort of reason. Or why is it that the different classes of predaceous animals, although surrounded by the materials out of which the human race has manufactured so many implements of warfare, have never been able to avail themselves of any of them, in aid of the instruments of destruction with which nature has furnished them? Apparently, because they are incapable of forming such general notions of the qualities and relations of external things, as are essential to the processes of imagination and reasoning, by which men are led to the contrivance and guided to the use of artificial weapons." (p. 2.)

Fully admitting the *general* correctness of these remarks, we think that there is not enough allowance made for the intellectual differences between the various classes and orders and genera and species of the lower animals, which, we are convinced, are as striking and as important as their corporeal diversities. We are of opinion that every kind of animal has its psychical as well as its physical powers, adapted to the kind of life which nature (in other words its Creator) intended that it should lead. There can be no doubt that this is the case in regard to the *instinctive* propensities; and this general law of adaptation to the natural habits of the animal appears to govern the hereditary transmission of *acquired* instincts; those being transmitted which have such a relation, and those being lost which are purely the result of human agency.* And if we attentively compare the *intellectual* faculties of each species with those of man, who possesses them all in the state of highest development, we think that, as in different individuals of the human race, we should find different faculties predominating in the several tribes; a view which was well stated by Gall, although, in working it out, he fell into many errors. Thus, in one individual the sensations are acute and the perceptions dull. In another, the intuitive perceptions are strong and ready; but there is a deficiency in the powers of reflection, owing to the want of the faculty of abstraction. And, in a third, the mind is capable of profound generalizations, but is not quick at observing. When the faculties of abstraction, or, in Hoffbauer's language, *intensity*, is wanting, silly imbecility is the result. When the perceptions are very slow, or the mind is deficient in *extensity*, the power of bringing itself into relation with external things, stupid imbecility or idiocy displays itself. Now the intellect of the monkey we regard as peculiarly deficient in *intensity ;* and that it should be so, the habits of the animal, and the circumstances in which it exists, afford quite sufficient reason. Of what use, for example, would be the power of making a fire to an animal that lives only in warm latitudes? And, to refer to Dr. Alison's other instance, of what use would the power of constructing weapons of offence be to animals already supplied by nature with all that is necessary for their support, and physically incapable of using them, if they were put (we were going to say "into their hands,") into their possession? And yet in their mode of lying in wait for their prey, and in the variety of stratagems they use to obtain it, these animals show a high amount of reasoning power, its limited scope being adapted to their peculiar circumstances. We shall presently endeavour to exhibit a little more in detail the gradation that may be traced in the different classes of animals in regard to the development of their intellectual powers, and the subordination to them of the instinctive.

There is one remarkable deficiency in the psychical powers of the lower animals, which we do not see expressly noticed by Dr. Alison. It is that of regulating or controlling the succession of thought. Metaphysicians differ as to the mode in which this power is exercised; but all agree that man possesses it by a direct or indirect operation of the will. "It may not be going too far to assert," it is justly remarked by Dr. Abercrombie, "that our condition in the scale both of moral and intellectual beings is, in a great measure, determined by the control which we have acquired over the succession of our thoughts, and by the sub-

* See Carpenter's Principles of Physiology, § 550, and the authorities there referred to.

jects on which they are habitually exercised." As far as we can infer the psychical powers of the lower animals from their actions, we do not think that there is any reason to believe in their possession of this faculty. And they thus stand in relation to those weak-minded persons of the human race who are deficient in it, all their trains of thought being obviously dependent on, and entirely governed by, either present sensations or the memory of past.

With respect to the *moral feelings* of animals, we have always regarded it as difficult to distinguish those exhibited by the highest among them from those of man; and have been accustomed to consider *his* possession of a notion of a spiritual being, in the light of an intuitive perception, immediately suggested to his mind by external occurrences without any act of reasoning, as the peculiar characteristic of his moral faculties, as the power of directing his current of thought is of his intellect. We are much pleased to find Dr. Alison entertaining a similar view.

"Although many of the lower animals are susceptible of the emotions of joy, and to a certain degree of gratitude and attachment, founded on the sense and recollection of benefits, none of them seem capable of forming the slightest notion of that Divine Power which has suggested itself to the human intellect in all ages and even in the rudest conditions of human existence; we should regard any act of praise or prayer as an infallible indication of a mental capacity of the same rank as our own." (p. 2.) The attachment of the dog to man, however, is evidently influenced by feelings of parallel character; but, like those of the young child, they are not susceptible of relation with any being not the object of sense,—"Man," it was expressively said by Burns, "is the god of the dog."

Having thus pointed out the existence, in many of the lower animals, of psychical endowments corresponding to those which we term the intellectual powers and moral feelings of man, we shall proceed to enquire what are the characteristics of those actions which are purely instinctive. These are well laid down by Dr. Alison; but to avoid confusion, we shall slightly modify his language, where it implies the participation of the will in actions purely instinctive. In all cases, those actions which are entitled to the appellation of instinctive, are generally understood to be characterized by two marks, quite sufficient to distinguish them from the effects of voluntary power guided by reason: 1. That, although in many cases experience is required to give the will command over the muscles concerned in its operations, no experience or education is required in order that the different actions which result from an instinctive impulse may follow one another with unerring precision. 2. That these actions are always performed by the same species of animal nearly, if not exactly in the same manner; presenting no such variation of the means applied to the object in view, and admitting of no such improvements in the progress of life or in the succession of ages, as we observe in the habits of individual men, or in the manners and customs of nations adapted to the attainment of any particular ends by those voluntary efforts which are guided by reason. A third distinctive mark, naturally resulting from the last, is at least equally characteristic, although much less generally observable: that these instinctive actions are seen to be performed in circumstances which reason informs us to be such as to render them nugatory for the ends which are usually accomplished by them, and for which they are obviously designed. The efforts made by migratory birds, even when confined, at their usual period of migration, the mistake of the flesh-fly, who deposits her eggs on the carrion plant instead of a piece of meat, or of the hen who sits on a pebble instead of an egg, are so many proofs that an instinctive action is prompted by an impulse, which results merely from a particular sensation or emotion being felt, not by anticipation of the effect which the action will produce.

If, with these views, we take a general survey of the habits of the different tribes of animals—distinguishing those actions which are invariably performed by all the individuals of one species under the same circumstances from those which exhibit a difference of physical character amongst them, and separating those which show a highly-refined adaptation of means to ends, such as we can scarcely conceive to exist among the lower classes, from those which give evidence of reasoning processes of a simple character—we cannot avoid the conclusion that the instincts are most remarkable, and most unaffected by any reasoning processes in the class of insects, and preeminently so in the order hymenoptera, which includes most of those that live in perfect societies, such as the bees, wasps, and ants. Now it is a little remarkable that, of all animals, insects are most remarkable for their *locomotive* powers, estimating these according to their size; and that, of all insects, the hymenoptera possess these powers in the highest degree. These facts we shall presently turn to further account. There can scarcely be a more extraordinary instance of the adaptation of means to ends, without (as there is every reason to believe) any *intention* on the part of the animal, than the peculiar construction of the comb of the bee. The substitution of the hexagonal form of the cell for the circular, by which a great deal of material is saved, whilst the purpose of the cell is equally answered, is but a comparatively simple example of this. It is in the mode in which the cells on the opposite side of the comb bear upon one another, that we see the most refined contrivance. The two sets of cells are so arranged that the centre of the bottom of one cell is opposite the point at which the partitions of three cells meet on the other side; and consequently the bottom of each cell is opposite a portion of the base of three cells opposed to it, instead of directly meeting the base of one, an arrangement which obviously adds great strength. But even this is not all. The bottom of the cell is not a flat partition, but is formed by the union of three planes at a certain angle; each of these planes forming part of the base of one of the opposite cells. The human mathematician has discovered, what would not be at once apparent to the mere observer, that, by the meeting of these planes at an angle, greater strength, in proportion to the amount of material employed, is obtained than if they formed part of one continuous surface. The determination of the particular angle at which these objects should be most effectually fulfilled, was long ago attempted by Maraldi. It is a problem requiring the highest powers of the mathematician to solve. The result obtained by him differed by only two minutes of a degree from the result of the admeasurement of the angle actually employed by the bee; and this approximation has been usually considered quite sufficiently close; the chances of error in the admeasurement of an angle made by so small a surface being considerable. More recently, however, Lord Brougham has retraced Maraldi's ground; and, by the application of still higher modes of investigation, he has determined that the bees were right and the mathematician wrong, having brought the result of his calculations to agree perfectly with the actual admeasurement!

No one, we think, will venture to say that the bees go through such a calculation. It is evident that the operations by which this remarkable effect is produced must be independent of any judgment or foresight on their parts, but must be as necessary a result of their physical conformation, as their mode of obtaining their food, or any other purely instinctive action. The same may be said of their other habits, in which the utmost foresight and contrivance is exhibited; but it can scarcely be regarded as *their* foresight and contrivance. It is in the *deviations* of the instincts of insects, and their accommodation to circumstances, as Mr. Spence justly remarks, that the exquisiteness of these faculties is most decidedly manifested. The instincts of the larger animals seem to undergo but slight modifications; or, when modified, it is rather by their reasoning powers. But insects (as is well known to those who have observed their habits, especially those of the social tribes,) often exhibit the most ingenious resources, their instincts surprisingly accommodating themselves to the new circumstances under which they are placed, in a manner even more wonderful and incomprehensible than their ordinary operations. But, it will be asked, do not these variations indicate the existence of reason in the insect tribes, and in what do they differ from those which the higher animals exhibit? We shall answer this enquiry in Dr. Alison's words:

"When such facts are duly considered, we cannot be surprised to find so intelligent a naturalist as Mr. Spence acknowledging that he had at one time arranged them as indications of reason in these animals. But, on further consideration, we shall probably see cause to acquiesce in his later and more matured judgment, which ascribes them to strictly instinctive, though singularly varying propensities; chiefly on two grounds, which exactly correspond with what have been previously stated as the most distinctive characters of instinct: 1. That, although various contrivances are fallen on by *all* bees, to enable them to continue their usual operations under varying external circumstances, yet there is no such variety observed, either in the conduct of individuals of the species, or in the conduct of different communities, as we cannot doubt must occur if the inhabitants of every hive were guided, on such unusual occasions, by processes of reasoning, by observation of the laws of nature, by experience, and by the anticipation of the effects of their actions. If such mental processes were their guide, we should certainly observe a difference in the conduct of experienced workers, and of those just emerged from their pupæ; and we should observe some variety in the expedients adopted in different hives, for meeting such accidents or difficulties. 2. While the varying operations of these animals for one particular end, the preservation of their own lives and the perpetuation of their species, are planned and combined in such a manner as to indicate consummate intelligence as to what is essential for that purpose, all these indications of instinct are limited to that object, and we see no evidence of the exercise of their senses suggesting to them any other trains of thought, or exciting them to the prosecution of other objects, such as a number of human intellects capable of planning and executing such works would certainly, sooner or later, attempt to accomplish. The degree of uniformity seen in their operations, and the limitation of the objects on which their faculties are exerted, are therefore our reason for thinking (though we do not wish to express ourselves with absolute confidence on the subject) that the mental processes concerned, even in those the most elaborate and artificial of the works of animals, belong to the same class as those notions of man which are prompted by his instinctive propensities as distinguished from his reason." (p. 21.)

At the same time it must be admitted that many acts of individuals and of communities among insects appear to indicate the guidance of some amount of reasoning power. But we must take leave to doubt some of the cases upon which this assertion rests. The narration of them is often coloured by the preconceived views of the individual, however desirous he may be of stating the bare facts; and in many instances we feel confident that a fuller knowledge of the natural habits and instincts of the species would show *that* to be a part of them which has been regarded as evidencing the operations of a reasoning power. Dr. Darwin's anecdote of a wasp is a case precisely in point. We shall first quote his own account of it, (Zoonomia, vol. i. p. 263:) "A wasp, on a gravel walk, had caught a fly nearly as large as himself; kneeling on the ground, I observed him separate the tail and head from the body part, to which the wings were attached. He then took the body part in his paws, and rose about two feet from the ground with it; but a gentle breeze wafting the wings of the fly turned him round in the air, and he settled again with his prey on the gravel. I then distinctly observed him cut off with his mouth, first one of the wings, and then the other, after which he flew away with it unmolested by the wind." Now on this we remark that, as we have been informed by two competent authorities, it is a *constant* habit of the wasps to cut off the head and wings of insects which they attempt to carry through the air. Even supposing (which we are disposed to question) that the wasp did really rise into the air without separating the wings, its alighting again to do so is no proof that it foreknew that its flight would be thus rendered easier, since it merely acted in accordance with its usual instinct, which some circumstance had at first caused it to forego. But, by subsequent narrators, this story, already quite good enough, has been further embellished. Dr. Alison quotes it, we are sorry to say, not from the original, but from Mr. Duncan's Tract on Instinct, where it is thus related: "Dr. Darwin observed a wasp with a large fly, nearly as big as itself. *Finding it too heavy*, it cut off the head and abdomen, and then carried off the remainder, with the wings attached to it, into the air; but *again* finding the breeze act on the wings, and impede its progress, it descended, and deliberately cut off the wings." Here we are given to understand that the wasp made *two* attempts; and it is obvious that this, were it the fact, would have an important influence on the view we take of the question on which it bears.

It would occupy too much space if we were to follow Dr. Alison through his very interesting review of the different groups of instinctive actions. He considers that they may be best classified under the following heads, according to their final causes:

1. Instincts designed for the preservation of the individuals exhibiting them. In this division are comprised those which relate to their means of escaping or repelling injury or violence; those by which food is procured; those which lead to construction of habitations; and those connected with hybernation.

2. Instincts for the propagation and support of offspring. Under this head may be placed, besides the mere sexual propensity and those immediately connected with it, those migrations of animals which are performed in furtherance of the object of propagation: the choice of a suitable place for depositing their eggs, nidification, incubation, and the nourishment of the young.

3. Instinctive propensities, the object of which is the advantage of the race, or of the animal creation generally, rather than of the individual or his progeny; together with some of which the object is still obscure. Among these may be mentioned the instinct of *congregation*, the *attachments* of animals to man, the tendency to *imitation*, &c.

We shall now offer a few remarks on the correspondence between the instincts of animals, and those tendencies to particular modes of action in the human mind, which go under the name of animal propensities, passions, and moral feelings. In regard to some of these there can be no kind of doubt. That the tendency to obtain food is an *instinct* in man, and parallel in its nature to that which prompts so many of the actions of the lower animals, will, we think, be generally admitted. If this be granted, we may take it as the basis of our enquiries into the nature of instinctive actions in general. If we analyze the train of actions to which it gives rise in adult man, we see that, like an emotion of the mind, it operates upon the reason, and sets it to work to provide the means of gratification. The present sense of hunger, caused by the condition of the stomach and of the corporeal system at large, acting upon the reason, and thus through the will, gives rise to those movements which convey into the stomach the food placed before the individual; and the anticipation of his future wants causes him to exert his whole psychical powers to provide the means of supplying them. But is it so in the infant? Very far from it. The intellectual powers are not yet developed by active employment. Experience has not given to the mind any data upon which to work. And, if the Creator had not provided a much more ready and unerring mode of supplying the wants of the corporeal system, it must speedily perish. The infant is purely under the guidance of instinct. When the system requires food, the contact of the nipple, or of any soft substance resembling it, with the lips is sufficient to occasion the action of sucking; and when the stomach is satisfied this action ceases. Now it has been sufficiently proved by observation and experiment, that the *brain* is not necessary to this action; but that it is one of those denominated by Dr. M. Hall, excito-motor, or reflex, depending upon the spinal cord and its nerves only. Can we, then, call such an action *voluntary?* Have we not sufficient evidence that all *voluntary* action originates in the brain? On this point we feel entirely satisfied. Now, if there are any animals whose instincts predominate as completely over the reasoning powers as they do in the human infant, we think it is equally evident that the actions prompted by them are involuntary. This is especially the case in insects; and there it is observed that many of the actions peculiar to the species are performed after the cephalic ganglia have been removed, as perfectly as they can be expected to do when no longer guided by the special sensations transmitted through them. But, we again repeat, in proportion as the reasoning powers predominate, will the actions stimulated by the instinctive propensity become of a voluntary character. It is quite possible even by them, however, for the propensity to exist in such an exaggerated form as, for the time at least, to overcome the will, even where that is strong and formed upon the basis of a sound judgment. This has been the case in nymphomania and satyriasis; perhaps also in bulimia: the stimulus arising from the excited state of the generative or digestive system overpowering all voluntary efforts to control the actions, just as the respiratory stimulus will do, when temporarily checked in its operations. This view we deem of some importance in regard to the philosophy of insanity; since it is evident that it applies to many other propensities besides those we have quoted.

The connexion of the passions and emotions with the instinctive propensities is sufficiently apparent. The lower kinds of the former, such as anger, fear, &c. border very closely upon the latter, and, like them, can operate immediately upon the corporeal system, without the intervention of the will. One of the instances quoted by Dr. Alison, as an example of the instinct of self-preservation, will show how closely the two classes are blended. We refer to the discharge, by the cuttle-fish, of the contents of its inkbag, when pursued; by which the water around it is so deeply coloured, that it escapes under a cloud. Now it will scarcely be doubted that this is an instinctive rather than an intelligent action; for if the animal had to learn it, the power would be of little use to him. From a comparison of the position and contents of its inkbag with the organs of other animals, we learn that it is in effect its urinary bladder. Now we know well that the discharge of this secretion, under the influence of fear, is a very common occurrence with many animals, and even with man; and personal experience may have assured some of our readers that the relaxation of the sphincters in this condition is completely involuntary.

The higher moral feelings would seem referrible to the same class; but they do not seem to have any *direct* power over the corporeal system. We might call them *mental instincts.* A closer analysis of these, distinguishing the different parts of the *morale,* according as they have or have not any immediate influence on the nerves, is much wanting. The deficiency will not long, we hope, remain unsupplied. The physician and metaphysician have worked in too great ignorance of each other's researches; and we do not regard it as a trifling benefit of the study of phrenology that it has tended to bring them together. But we are compelled to remark that, among the professed cultivators of the latter science, there are very few who give due weight to the results obtained by recent physiological investigations on the nervous system at large; and most of them seem to take it for granted that the brain does many things which are the real province of the spinal cord.

Rigidly maintaining, then, the essential superiority of the *intellect* of man over that of all other animals, it can scarcely be denied that the greater number of the *active* powers of the human mind, which furnish the chief motives to action, are on the same footing with those which operate on the lower animals. Not only are our appetites similar to theirs, but the greater number of the desires of which we are conscious are either shared with us by them, or at least would seem to belong to the same class as their instincts. "It is interesting," remarks Dr. Alison, "to reflect on the different powers, to the operation of which we can trace the unceasing changes continually taking place around us, and particularly on the gradation, and very gradual transition that may be observed, from those by which inanimate matter is continually moved and changed, up to those which emanate from the intellect of man."

We shall follow Dr. Alison's account of this gradation, with some modifications and additions of our own.

By the original impulse given to the world, and by the laws of gravitation and of motion impressed on all matter, the greater and more striking movements of the inanimate world around us are continually determined; and by the laws of chemistry, those movements are made subservient to constant changes in the composition of the organic world. Again, by the laws which were impressed on the lower class of living beings at the time of their introduction into the world, a constant succession of living vegetable structures is determined, merely by the agency of air and water, heat and light, on those already existing. The organized structures into which these elements are converted are found to exhibit properties peculiar to themselves; and among these is the power of contracting upon the application of a stimulus. In this manner are the sensible motions produced which are occasionally observed in plants. We shall, then, regard vegetables as peculiarly, but not entirely, constituting *the kingdom of organic life.* In immediate but still obscure connexion with the lowest of the vegetable creation are the lowest of the animal tribes. Here we see indications of the same simple contractility which plants enjoy, but having a still more important relation to the well-being of the individual; and in addition to the movements thus produced, we witness others which appear to be consequent upon sensations, although none which clearly evidence intelligence and design on the part of the animals themselves. Proceeding still higher, we observe these instinctive movements becoming more complex in their character, and more refined and special in their objects; until we arrive at the class of insects which, as already observed, seem to possess the highest amount of instinctive or intuitional faculties, of any animals known. But we by no means find *intelligence* increasing in the same ratio. On the contrary, it remains very low. On the other hand, we observe those motions which are produced only by the direct contractility of the tissues stimulated, bearing a smaller and smaller proportion to the whole; and at last restricted to the organic functions, with which they always remain associated. Ascending from the class of insects through the vertebrated series, we observe a gradually increasing development of the reasoning powers, or intelligence; and a gradually fading away of the instincts, which become subordinate to the higher psychical faculties; until in man, the pure uncontrolled operation of instinct is only seen in infancy, when these faculties are still dormant.

Now it is evident that the whole class of instinctive actions has for its sole object the maintenance of *animal life;* and this in combination with the power of locomotion, also peculiar to animals. It will be recollected that, in the hymenopterous insects, *both* these powers are developed in the highest degree. Hence we should be led to regard these as typical of the *kingdom of animal life;* and to regard *man's* place in that kingdom as not being at its head or in its centre, but as being at one of the extremes; at the point, in fact, at which we may believe it to touch another kingdom, one of *pure intelligence.* And as the infant, in its progress to the vigour of manhood, exhibits those phases of psychical development which may be traced in ascending the animal scale, so do we find that, in the still further progress of years, whilst the body decays, the spirit, rightly disciplined and steadily elevated, approaches nearer and nearer to the type which we presume to consider as characterizing more exalted beings.

Let not these speculations be regarded as misplaced. They tend to show the true nobility of man's rational and moral nature; and the mode in which he may most effectually fulfil the ends for which his Creator designed him. From these alone he might learn the evil of yielding to the "fleshy lusts which war against the soul;" and the dignity of those pursuits which exercise the intellect, and expand and strengthen those lofty moral feelings which are peculiar to man.

Many other such thoughts are suggested to us by the concluding passages of Dr. Alison's very interesting article, in which the important question of "free will" is briefly but clearly treated. There are also some remarks upon Mr. Whewell's views in regard to the use of the study of final causes in physiology, which harmonize closely with those which we formerly made upon the same subject, (See Vol. V. p. 338, et seq.;) and also upon the value of the phenomena of instinct, as indications of design in the universe.

We have but a few words to say in conclusion on the two other books at the head of this article. If our readers should look into Dr. Bushnan's Treatise, with an expectation of finding the *philosophy* of the subject which it professes to unfold, we fear they will be disappointed. It contains much that is clever, but not much that is original. Often as the author has professedly quoted from the writings of his preceptor, Dr. Fletcher, we think that he has hardly sufficiently acknowledged his obligations to them; for the great bulk of his book seems to us elaborated from certain parts of Dr. F.'s Physiology, in which his peculiar opinions are set forth, with a very grievous mutilation of those arguments, by which that very clever writer often contrived to make the worse appear the better reason. If any of our readers wish to find a justification of our assertion, let them compare the corresponding chapters in the two works on Irritability and its Seat in Plants and Animals. The most original part of the book is that in which he undertakes to demolish Lord Brougham's arguments for the Immateriality of the Soul. On the distinctive attributes of instinct and reason, he expresses himself pretty clearly, and locates the former in the spinal cord, and the latter in the brain; in which we think he is not far from the truth. But he represents instinctive actions as always depending on sensation, which is incorrect, in the extended sense at least in which he uses the term instinct. He is a strenuous advocate for the concession of reasoning powers to the lower animals; and quotes Darwin's wasp, with a still fuller account of both flights, and of the mental operations which passed in its cephalic ganglia, than that which we have already noticed. There are many acute remarks in the book; but we would rather say that its author was clever, than that it was good.

Mr. Swainson's Treatise contains a very good compilation of the materials furnished by several authors, in regard to the habits and instincts of animals, arranged under their respective heads; and it is, therefore, a very useful work to those who stand in need of such information. Of the value of his general observations, however, we cannot say much. The following will serve as a specimen. "If we once allow the least degree of reason to the brute creation, we must concede a portion of it altogether incompatible with their situation."

